# The outcome and predictive factors of sunitinib therapy in advanced gastrointestinal stromal tumors (GIST) after imatinib failure - one institution study

**DOI:** 10.1186/1471-2407-12-107

**Published:** 2012-03-22

**Authors:** Piotr Rutkowski, Elżbieta Bylina, Anna Klimczak, Tomasz Świtaj, Sławomir Falkowski, Jacek Kroc, Iwona Ługowska, Magdalena Brzeskwiniewicz, Wojciech Melerowicz, Czesław Osuch, Ewa Mierzejewska, Kacper Wasielewski, Agnieszka Woźniak, Urszula Grzesiakowska, Zbigniew I Nowecki, Janusz A Siedlecki, Janusz Limon

**Affiliations:** 1 Department of Soft Tissue/Bone Sarcoma and Melanoma, Maria Sklodowska-Curie Memorial Cancer Center and Institute of Oncology, Warsaw, Poland; 2 Pfizer Poland, Warsaw, Poland; 3 Department of Biology and Genetics, Medical University of Gdansk, Gdansk, Poland; 4 GreaterPoland Oncological Center, Poznan, Poland; 5 Department of General Surgery, Jagiellonian University, Medical Faculty, Cracow, Poland; 6 Department of Biostatistics, Institute of Mother and Child, Warsaw, Poland; 7 Laboratory of Experimental Oncology and Department of General Medical Oncology, KU Leuven and University Hospital, Leuven, Belgium; 8 Department of Radiology, Maria Sklodowska-Curie Memorial Cancer Center and Institute of Oncology, Warsaw, Poland; 9 Department of Gastrointestinal Tumors, Maria Sklodowska-Curie Memorial Cancer Center and Institute of Oncology, Warsaw, Poland; 10 Department of Molecular Biology, Maria Sklodowska-Curie Memorial Cancer Center and Institute of Oncology, Warsaw, Poland

**Keywords:** Sunitinib, Genotype, GIST, Prognosis, Predictive factors, Arterial hypertension

## Abstract

**Background:**

Gastrointestinal stromal tumors (GIST) mutational status is recognized factor related to the results of tyrosine kinase inhibitors therapy such as imatinib (IM) or sunitinib (SU). Arterial hypertension (AH) is common adverse event related to SU, reported as predictive factor in renal cell carcinoma. *The aim *of the study was to analyze the outcomes and factors predicting results of SU therapy in inoperable/metastatic CD117(+) GIST patients after IM failure.

**Methods:**

We identified 137 consecutive patients with advanced inoperable/metastatic GIST treated in one center with SU (2^nd ^line treatment). Median follow-up time was 23 months. Additionally, in 39 patients there were analyzed selected constitutive single nucleotide polymorphisms (SNPs) of *VEGFA *and *VEGFR2 *genes.

**Results:**

One year progression-free survival (PFS; calculated from the start of SU) rate was 42% and median PFS was 43 weeks. The estimated overall survival (OS, calculated both from start of SU or IM) was 74 weeks and 51 months, respectively. One-year PFS was 65% (median 74 weeks) in 55 patients with AH *vs*. 22% (median 17 weeks) in patients without AH. Patients with primary tumors carrying mutations in *KIT *exon 9 or wild-type had substantially better 1-year PFS (68% and 57%; median 65.5 and 50.5 weeks, respectively) than patients having tumors with *KIT *exon 11 or *PDGFRA *mutations (34% and 15%; median 36.8 and 9 weeks, respectively). We identified two independent factors with significant impact on PFS and OS in univariate and multivariate analysis: primary tumor genotype and presence of AH. The most common adverse events during therapy were: fatigue, AH, hypothyroidism, hand and foot syndrome, mucositis, skin reactions, dyspepsia, and diarrhea. Two deaths were assessed as related to tumor rupture caused by reaction to SU therapy. The presence of C-allele in rs833061 and the T-allele in rs3025039 polymorphism of *VEGFA *were associated with significantly higher risk of hypothyroidism (OR: 10.0 p = 0.041 and OR: 10.5; p = 0.015, respectively).

**Conclusions:**

We confirmed that many advanced GIST patients benefit from SU therapy with OS > 1.5 year. Primary tumor *KIT/PDGFRA *genotype and SU-induced AH, as surrogate of its antiangiogenic activity are two independent factors influencing both PFS and OS.

**Note:**

The preliminary data of this study were presented during Annual Meeting of American Society of Clinical Oncology, 4-8 June 2011 and Connective Tissue Oncology Society Meeting, 26-28 October 2011 in Chicago, IL.

## Background

Unprecedented improvement in advanced gastrointestinal stromal tumors (GIST management has been achieved due to recent recognition of the important biological role of activating mutations in *KIT *and *PDGFRA *(platelet-derived growth factor receptor- alpha) genes. Those observations led to the introduction of imatinib mesylate, a small-molecule selective inhibitor of the receptor tyrosine kinases such as stem-cell factor receptor (KIT, CD117), BCR-ABL and platelet-derived growth factor receptors (PDGFRs)-A and -B. Imatinib revolutionized the outcome of patients with advanced CD117-positive GISTs and is currently approved as the first-line treatment in advanced (metastatic and/or inoperable) GISTs [1-5]. However, the spectacular response to imatinib therapy is time-limited and secondary resistance to imatinib therapy (after initial stabilization or response) develops in majority of patients [4].

Currently, the only approved second-line drug is sunitinib malate - a multitargeted agent, an inhibitor of tyrosine kinase, of KIT and PDGFRA/B and of the vascular endothelial growth factor receptors (VEGFRs)-1, -2 and 3, FMS-like tyrosine kinase-3 (FLT3), colony stimulating factor 1 receptor (CSF-1R), and glial cell-line derived neurotrophic factor receptor (REarranged during Transfection; RET) [6-11]. Sunitinib possesses both antiangiogenic and cytostatic properties and by competing with ATP binding prevents multiple receptor tyrosine kinases phosphorylation *in vitro *and *in vivo*. Two phase II, one phase III and one "treatment-use" trials have investigated the activity of sunitinib in GIST patients after the failure of prior imatinib treatment, and all these trials have shown the significant activity of sunitinib in this population of patients [11-14]. The objective clinical benefit was achieved in approximately 60% of GIST patients who received sunitinib after failure of prior imatinib treatment [11-14]. Median progression -free survival time on sunitinib is 6-8 months. The adverse events reported during this therapy are frequent. The most common treatment-related adverse events were fatigue, diarrhea, skin discoloration, nausea, mucositis, arterial hypertension, hand and foot syndrome (palmar-plantar erythrodysesthesia), impairment of left ventricular ejection fraction and hypothyroidism [12,14].

Moreover, arterial hypertension was not only the common adverse event during sunitinib therapy, but it was reported as predictive factor for results of renal-cell carcinoma (RCC) patients [15,16]. This phenomenon has not been yet analyzed in GIST patients.

There is a lack of studies analyzing the outcome of sunitinib in advanced GISTs after imatinib failure therapy in routine practice outside clinical trials. Thus, the aim of our study was to evaluate factors predicting results and toxicity of SU second-line therapy in inoperable/metastatic GISTs. Additionally, we have investigated the impact of the selected single nucleotide polymorphisms (SNPs) in *VEGFA *and *VEGFR2 *genes on sunitinib-related toxicity in the subgroup of patients.

## Patients and Methods

### Patients

We analyzed prospectively collected data of 137 consecutive patients treated with sunitinib maleate because of inoperable and/or metastatic CD117 positive GIST enrolled into therapy between October, 2005 and February, 2011, reviewed in one tertiary cancer center. All patients met the following criteria for sunitinib treatment: 1) histological diagnosis of GIST, confirmed by CD117-immunopositivity (DAKO; Carpintiera, CA), 2) metastatic and/or inoperable lesions after failure on prior treatment with imatinib (confirmed progressive disease or unacceptable toxicity) 3) measurable disease on computed tomography (CT) scans, 4) WHO performance status ≤3, 5) no concomitant therapy for disease, 6) adequate renal, cardiac and liver function.

Each patient provided informed consent for the study and collection of clinical and molecular data prior sunitinib therapy. The study had been approved by the local Bio-Ethics Committee according to Good Clinical Practice Guidelines (approvals from Bio-Ethics Committees from Medical University of Gdansk and from Maria Sklodowska-Curie Memorial Cancer Center and Institute of Oncology, Warsaw KB/9/2011 and approval for Polish Clinical GIST Registry by Internal Review Board 119/2002). Patients provided additional informed consent for taking the 5 ml blood samples for gene polymorphisms analysis. Patients did not undergo any further selection; 35 patients were initially included in the treatment-use trial A6181036.

All patients were treated with sunitinib in initial licensed dose of 50 mg daily in 6 weeks cycle (4 weeks on/2 weeks off therapy), however the dosing could be reduced (to 37.5 mg or 25 mg) or delayed or modulated to the dosing of 37.5 mg on continuous schedule to optimize the benefit-risk profile according to decision of treating physician. The treatment was continued until confirmed progression of the disease or unacceptable toxicity. All patients were followed carefully with median follow-up time of 23 months (range: 6-68 months). The objective response of GIST to sunitinib therapy was evaluated with serial CT examinations (performed every 2-3 months), according to Response Evaluation Criteria in Solid Tumors (RECIST) version 1.0 [17]. In case of progression, patients were treated with other different tyrosine kinase inhibitors or cytotoxic chemotherapy or best supportive care only. If possible, they were included into clinical trials with new compounds. Toxic effects were graded with National Cancer Institute common toxicity criteria, version 3.0 [18].

### Genotyping

Genomic screening was performed for the presence of the *KIT *(exons 9, 11, 13, and 17) or *PDGFRA *(exons 12, 14 and 18) genes mutation in randomly selected 89 cases, based on DNA isolated from paraffin-embedded or fresh frozen imatinib-naive tumor tissues, as previously described [19].

### SNPs analysis

Genomic DNA was isolated from peripheral blood samples of 39 consenting patients using the standard protocol with proteinase K digestion, phenol - chloroform extraction and ethanol precipitation. Three selected *VEGFA *SNPs: rs699947 (-2578 C > A), rs3025039 (+936 C > T), and rs2010963 (+405 G > C) and two *VEGFR2 (KDR) *SNPs: rs1531289 (3405-92A > G) and rs1870377 (+1416 T > A) were genotyped using restriction fragment length polymorphism (RFLP) method. The restriction enzymes which detected - SNPs mentioned above were *BsaIII*, *BsmF1*, *NlaIII *and *AluI*, respectively. Amplified DNA was digested with endonucleases overnight at optimal temperatures according to the manufacturer (Fermentas, Thermo Scientific) and then electrophoresed on 2% agarose gel. For identification of an additional *VEGFA *SNP: - rs833061 (460 T > C) direct sequencing was performed using ABI 3100 Genetic Analyser (Applied Biosystems Inc. Foster City, CA). Primers sequences and PCR cycling parameters are available on request.

### Statistical analysis

Contingency tables were analyzed by the chi-square test. Progression-free survival (PFS) time was calculated from the date of the start of sunitinib treatment to the date of the most recent follow-up, or progression or death due to the disease. Overall survival (OS) time was calculated either from the date of the start of imatinib and sunitinib treatment to the date of the most recent follow-up or death due to the disease [except cases of two patients, whose death was attributed to adverse event (AE) of sunitinib therapy]. PFS was assessed with respect to the following variables: demographic data (age at the diagnosis ≤ 45 or > 45 years; gender), primary tumor genotype (*KIT *11 exon, *KIT *9 exon, any *PDGFRA *mutations and wild-type cases), length of previous therapy on imatinib (≤ 6, > 6-12, > 12 months), and presence of arterial hypertension (defined as occurrence of systolic blood pressure > 140 mm Hg or diastolic blood pressure > 90 mm Hg, or deterioration of preexisting AH during the first three months of therapy with sunitinib). The Kaplan-Meier method was used for analysis of survival curves, compared by log-rank test.

For univariate comparison of the survival between groups, the Kaplan-Meier estimator was used with generalized Wilcoxon and the log-rank tests. For PFS/OS comparisons in relation to presence of arterial hypertension two cases with early death due to tumor perforation were excluded to minimize the lead-time bias of the results. In multivariate analysis of the factors associated with PFS and OS, we used Cox proportional hazards models, applying the stepwise model building procedure that included all covariates significant at 20% level in bivariate analysis. Two-way interactions were then considered in the model. Differences were considered statistically significant if p-values were < 0.05. These statistical computations were performed using Statistica 6.1 software [Statsoft^®^; Tulsa, OK].

The Hardy-Weinberg equilibrium analysis of *VEGFA *and *VEGFR2 *polymorphisms was performed using a chi-square test with one degree of freedom. Multiplicative, dominant and recessive models were tested in every SNP for the associations with OS, PFS and treatment AEs. Odds ratios (ORs) with their exact 95% confidence intervals (CI) were calculated and the exact test for OR was used. The polymorphisms analyses were performed using StatXact-3 software, version 3.1.

## Results

### Clinicopathological data

The distribution of clinical and pathological data of patients included in the study is listed in Table 1. There were 74 male and 63 female patients, with median age at the start of sunitinib therapy 55 years (range: 15 - 82). The majority of primary tumors were located in the small intestine (57.6%), followed by the stomach (33.5%). All but two patients had documented progression on imatinib. Majority of patients (70.1%) were pre-treated with imatinib for more than one year. Almost 90% of patients started sunitinib therapy being in relatively good performance status (0-1).

**Table 1:** Characteristics of 137 patients treated with sunitinib due to advanced GIST

Clinicopathological features		No of patients (%)
Total number of patients		137 (100)

Age [years] at the start of therapy with sunitinib	Median (range)	55 (15-82)
	
	≤45	14 (10)
	
	> 45	123 (88)

Gender	Female	63 (46)
	
	Male	74 (54)

Primary tumor site	Stomach	46 (33.5)
	
	small bowel	79 (57.6)
	
	large bowel/rectum	4 (2.9)
	
	other or intraperitoneally with unknown primary origin	8 (5.8)

Time on imatinib therapy	≤ 6 months (early resistance)	25 (18.2)
	
	6-12 months	16 (11.7)
	
	> 12 months	96 (70.1)

Primary reason for stop of imatinib therapy	Disease progression	135 (98.5)
	
	Imatinib intolerance	2 (1.5)

ECOG Performance Status	0	48 (35)
	
	1	72 (52.6)
	
	≥2	17 (12.4)

Tumor genotype*	Exon 11 *KIT *mutation	52 (58.4)
	
	Exon 9 *KIT *mutation	15 (16.9)
	
	*PDGFRA *mutation	12 (13.5)
	
	Wild-type	10 (11.2)
	
	Data not available	48

* mutational status was evaluated in 89 cases (65%)

### Mutational analysis data

The distribution of patients according to the initial tumor mutational status is shown in Table 1. In the group of 89 patients, whose initial tumor mutational status was evaluated, 58.4% of GISTs had an exon 11 *KIT *mutation, 16.9% had an exon 9 *KIT *mutation, 13.5% had *PDGFRA *gene mutation (11 of 12 cases had D842V mutation) and in 11.2% of tumors we have not detected any mutations (wild-type).

### Treatment toxicity

Adverse events were common during sunitinib treatment (127/137 evaluated patients; 93%), and in 31.4% of patients they were assessed as grade 3/4 (Table 2). The most common non-hematological adverse events were: fatigue (65%), arterial hypertension (43%), hand-foot syndrome (40%), hypothyroidism (31%), skin/hair discoloration (30%), diarrhea (28%) and mucositis (25.5%). The frequency of reported hematological toxicity was as follows: anemia (37%), neutropenia (36%) and thrombocytopenia (13%). Two deaths (grade 5) due to the tumor rupture and hemorrhage were assessed as related to reaction to sunitinib therapy (during 1^st ^month of therapy). Additionally, three patients were operated due to tumor perforation (bleeding/bowel perforation) also attributed to reaction to sunitinib. For management of drug toxicity sunitinib dose was reduced to 37.5/25 mg in 44% of cases (60 patients).

**Table 2:** The most common adverse events (AEs) during sunitinib therapy in the entire analyzed group of GIST patients

	Any grade		Grade 3/4	
**AEs**	n	%	N	%

Any treatment-related AE	127	92.7	43	31.4

Fatigue	89	65	12	8.7

Diarrhea	37	28	4	2.9

Hand-foot syndrome	55	40	3	2.2

Decreased appetite/dysgeusia	25	18.2	0	0

Mucositis	35	25.5	2	1.5

*Hypertension*	*59*	*43*	*4*	*2.9*

Neutropenia	49	36	7	5.1

Anemia	51	37	8	5.8

Skin/hair discoloration	41	30	0	0

Dyspepsia	43	31.4	0	0

Thrombocytopenia	18	13.1	3	2.2

Hypothyroidism	42	31	2	1.5

### Outcomes of sunitinib treatment

Median PFS was 43 weeks and estimated 1-year PFS rate was 42%. Progression of disease during sunitinib therapy was observed in 105 cases (77%). At the time of the analysis, 45 patients (33%) were alive. Estimated 2-year OS rate was 40% and median OS was 73.5 weeks. Estimated 5-year OS in this group of patients was 45% (when calculated from the date of imatinib start) and median OS - 51 months.

The best responses observed during sunitinib therapy and estimated by CT imaging (two consecutive examinations) according to RECIST criteria were as follows: none complete responses (CRs), 21 (15%) partial responses (PRs), 62 (45%) stable disease (SD) at least four months, 51 (37%) progressive disease (PD) and three patients were not assessable for response. Overall clinical benefit of sunitinib therapy (counted as the sum of CR, PR and SD rates) was 60%.

### Correlations between mutational status of primary GISTs and response to sunitinib therapy

We have found a strong relationship (p < 0.001) between the primary tumor genotype and best observed, confirmed response to sunitinib according to RECIST criteria: the best outcomes were observed for *KIT *exon 9 mutants (six PR - 40%, seven SD - 47%, two PD - 13%), followed by wild-type GISTs (seven SD - 70% and three PD - 30%) and *KIT *exon 11 mutants (10 PR - 19%, 23 SD - 44%, 19 PD - 37%); the worst results of sunitinib therapy were found in patients with *PDGFRA *mutated GISTs (two SD - 17% and 10 PD - 82%).

### Factors influencing PFS and OS during sunitinib therapy

**Figure 1:**
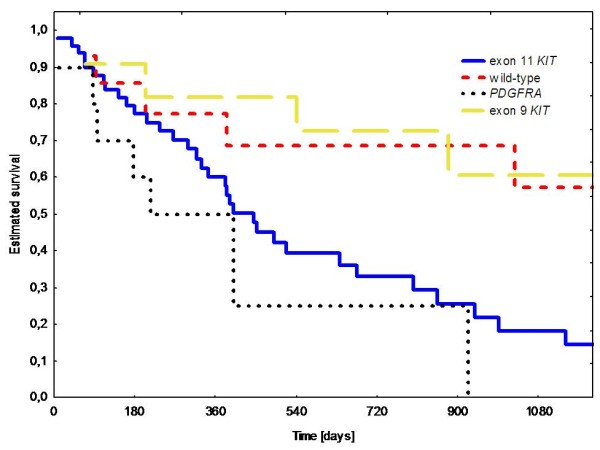
**Overall survival during sunitinib therapy according to primary tumor mutational status**.

**Figure 2:**
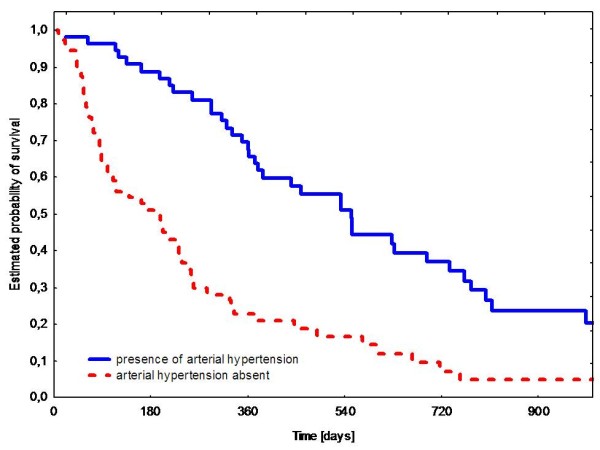
**Progression-free survival during sunitinib therapy according to presence of arterial hypertension**.

In univariate analysis two factors significantly correlated with shorter PFS and OS that were: tumor genotype: exon 11 *KIT *or *PDGFRA *mutation (p = 0.04 and p = 0.04, respectively) (Figure 1), and absence of arterial hypertension during sunitinib therapy (p = 0.0001 and p = 0.001) (Figure 2). We did not find any significant correlation between PFS/OS and patients gender and age at diagnosis, GIST primary tumor location, WHO performance status 0 *vs*. 1, or time on previous imatinib treatment. Estimated 1-year PFS and 2-year OS according to primary tumor genotype were as follows: *KIT *exon 9 mutations - 68%/73% (median 65.5/151.5 weeks), wild type - 57%/70% (median 50.5/121 weeks), *KIT *exon 11 mutations - 34%/34% (median 36.8/65.5 weeks) and *PDGFRA *mutations - 15%/25% (median 9/40 weeks). Patients with presence of arterial hypertension during first three months of sunitinib therapy had substantially better 1-year PFS and 2-year OS than patients without this adverse event (65% *vs*. 22%, and 63% *vs*. 18%; median 77 *vs*. 25.5 weeks, and 128.5 *vs*. 43 weeks, respectively).

In the multivariate analysis two factors (tumor genotype and arterial hypertension) had independent predictive value for PSF and OS (Tables 3 and 4).

**Table 3:** Multivariate analysis of prognostic factors for progression-free survival

	Hazard ratio	95% CI	Standard Error	z	p value
Arterial hypertension Yes	0.2025	0.1097 - 0.3739	0.0633	-5.11	0.000

Primary tumor mutation Wild type	0.3292	0.1447 - 0.7491	0.1381	-2.65	0.008

*PDGFRA *mutation	1.8753	0.9096 - 3.8661	0.0692	1.70	0.049

**Table 4:** Multivariate analysis of prognostic factors for overall survival

	Hazard ratio	95% CI	Standard Error	z	p value
Primary tumor mutation Exon 9 *KIT *mutation	0.7491	0.3308 - 1.6968	0.3125	-0.69	0.489

*PDGFRA *mutation	1.2678	0.5487 - 2.9327	0.5425	0.55	0.579

Wild-type	0.1029	0.0317 - 0.3336	0.0618	-3.79	0.000

Arterial hypertension Yes	0.2056	0.1006 - 0.4201	0.0749	-4.34	0.000

*CI *confidence interval

### Genetic variations of VEGFA and VEGFR2 genes

The genotype frequency of *VEGFA/VEGFR2 *gene SNPs are presented in Table 5. All analyzed genotypes were in Hardy-Weinberg equilibrium. The higher risk of hypothyroidism during sunitinib therapy was associated with: C-allele in rs833061 (OR: 10.l; p = 0.041) and T-allele in rs3025039 (OR: 10.5; p = 0.015) (Table 6). We did not find the correlation between the presence of SNPs and arterial hypertension, hand-foot-syndrome, skin toxicity/mucositis or diarrhea (data not shown).

**Table 5:** The genotype frequency of *VEGFA/VEGFR2 *genes SNPs and Hardy-Weinberg equilibrium (HWE) test results

*Polymorphism*	*rs number*	*Genotype (%)*			*HWE exact p value *	*Frequency of risk allele*
				
		*Homozygous (wild) *	*Heterozygou*	*Homozygous (mutant)*		
***VEGFA***						

-2578 C > A	rs699947	32.4	54.1	13.5	0.73	0.41

-460 T > C	rs833061	29.7	45.9	24.3	0.74	0.47

936 C > T	rs3025039	75.7	24.3	-	1.00	0.12

405 G > C	rs2010963	24.3	40.5	35.1	0.32	0.55

***VEGFR2 (= KDR)***						

3405-92A > G	rs1531289	48.6	45.7	5.7	0.69	0.29

1416 T > A	rs1870377	53.1	40.6	6.3	1.00	0.27

**Table 6:** Relationship between *VEGFA/VEGFR2 *gene polymorphisms and sunitinib-induced hypothyroidism grade ≥2.Statistically significant correlations are marked in bold

Polymorphism	Reference/risk genotype	*OR*	*95%CI*	*p *value
***VEGFA***				

rs699947 (C > A)	CC → AC → AA	2.38	0.82 - 6.99	0.125

rs833061 (**T > C)**	**TT *vs *CT + CC**	**10.00**	**1.07 - 466.60**	**0.041**

rs3025039 (**C > T)**	**CC *vs *CT + TT**	**10.50**	**1.42 - 117.40**	**0.015**

rs2010963 (G > C)	GG *vs *CG + CC	1.29	0.22 - 9.62	1.000

***VEGFR2 (= KDR)***				

rs1531289 (A > G)	GG *vs *AG + AA	1.60	0.33 - 8.29	0.758

rs1870377 (T > A)	TT → AT → AA	0.74	0.19 - 2.64	0.822

*CI *confidence interval, *OR *odds ratio

## Discussion

A majority of patients with advanced GISTs ultimately stop responding to imatinib and unquestionably management of disease resistant to first-line treatment represents a clinical challenge [4]. Insights into resistance mechanisms have allowed developing several strategies in patients with progression during imatinib treatment. In case of generalized progression (or intolerance to imatinib) the main option is using monotherapy with alternative multi-tyrosine kinase inhibitor - sunitinib, which remains the only approved second line drug for the treatment of advanced GISTs after imatinib therapy failure [20]. Sunitinib has demonstrated robust clinical effectiveness in imatinib-resistant or -intolerant GIST as shown in randomized, placebo-controlled phase III trial in which the median time to tumor progression for patients treated with sunitinib was more than four times longer than that for patients receiving placebo (27.3 *vs*. 6.4 weeks) [12]. Present study, according to our best knowledge, represents of the largest series of GIST patients after imatinib failure analyzed for the outcome of sunitinib treatment in routine clinical practice outside randomized, controlled clinical trial.

We have also attempted to prove tumor genotype implications and to find new predictive factors in this group of patients. We have confirmed that many advanced GIST patients benefit from sunitinib therapy (mainly due to stabilization of disease according to RECIST, not Choi criteria [21]) with OS exceeding 1.5 years. The median PFS longer than seven months is almost equal to the results of the Korean one-institution study [22]. We have also confirmed in more detailed way and on the larger group of patients, than ever published, data regarding the correlation between primary tumor mutational status and sunitinib treatment outcomes [22-24]. As for imatinib, *KIT *mutation status appears to serve as a predictor of tumor response to sunitinib. We have proven that, contrary to imatinib, tumors initially (pre-imatinib treatment) bearing *KIT *exon 9 mutation or with wild-type genotype have a higher chance to respond to sunitinib. Moreover, GISTs harboring *KIT *exon 9 mutations appear to be more sensitive to sunitinib than those with primary *KIT *exon 11 mutations (however we have observed some objective responses also in this group of patients). The clinical benefit of sunitinib in wild-type cases is also clear. We have not observed any response to sunitinib in group of patients with *PDGFRA *mutations (mainly D842V), which has been also shown in preclinical data. We did not analyze the impact of secondary mutations, although patients from clinical trials with tumors harboring a secondary mutation in exon 13 or exon 14 *KIT *have a longer PFS than patients with exon 17 or 18 mutations [23,25-27]. On the other hand, utility of analysis of secondary mutations is very challenging because imatinib-resistant GISTs are very heterogeneous with multiple clones having different secondary mutations within the same or different nodules [28-30].

Sunitinib therapy is associated with several adverse events, which were generally mild to moderate and could be managed by dose modulation (including continuous administration of lower dose) [20,22,24]. The toxicity profile reported in our study is similar to that observed in clinical trials, with exception of hypothyroidism, which occurred in more than 30% of patients (it has been reported outside clinical trials [31,32]). However, up to one third of cases were classified as more severe toxicity (and two deaths due to tumor hemorrhage were classified as related to sunitinib therapy). Our own experience with patients with unresectable or metastatic GISTs, treated with tyrosine kinase inhibitors, suggested the higher incidence of emergency operations for gastrointestinal bleeding, bowel obstruction, or abscess, occur during second-line therapy with sunitinib than during first-line therapy with imatinib [33,34]. This increased incidence of complications leading to surgical interventions with sunitinib could be associated with the presence of more advanced and drug-resistant disease, or to the direct mechanism of action of sunitinib, i.e., the combination of cytotoxic and antiangiogenic activity, leading to dramatic tumor response.

Arterial hypertension is one of the most common complications of sunitinib therapy, occurring usually early after treatment initiation. Serial monitoring of blood pressure is recommended during therapy with sunitinib. Sunitinib-induced arterial hypertension may also serve as biomarker of antitumor efficacy (probably by antiangiogenic mechanism), because it was an independent factor influencing patient both progression-free and overall survival. Antiagiogenic activity may play an important role in therapy of sarcomas, what has been recently confirmed by positive results of phase III trial with pazopanib in pre-treated soft tissue sarcoma patients [35]. Similar relationships between arterial hypertension induced by VEGF inhibitors (including sunitinib) and oncological outcomes have been reported in renal cell carcinoma patients [15,16,36-39]. Treatment-induced persistent hypertension was associated with frequent tumor response, a long time to disease progression and longer overall survival [39]. Clinical outcomes are not compromised by treatment with anti-hypertension medications, moreover, patients who required at least three antihypertensive drugs had the longest PFS and OS [38]. There are proposed some hypothetical mechanisms leading to hypertension related to sunitinib, e.g. presence of less-perfused microvessels and/or diminished number of microvessels, decreasing nitric oxide production and activation of the endothelin-1 pathway leading to vasoconstriction [40,41].

In the subgroup of patients we have analyzed some possible pharmacogenetical relationships with sunitinib tolerance. It has been shown that single nucleotide polymorphisms of *VEGF *and *VEGFR2 *genes has some potential as biomarkers for clinical outcomes and toxicity of VEGF pathway targeted therapy [42-46]. We have not studied correlation between SNPs of *VEGFA/VEGFR *genes and outcomes of therapy due to limited number of cases, but we have found clear associations between two SNPs of *VEGFA *gene and sunitinib-induced hypothyroidism. The molecular mechanisms of hypothyroidism induced by sunitinib are unknown, but recent studies have suggested that VEGFR inhibition can induce vasculature regression in various organs, predominantly in thyroid, what can be linked to different properties of VEGF protein caused by gene polymorphisms and sunitinib sensitivity [47,48].

## Conclusions

To summarize, we confirmed that many advanced GIST patients benefit from sunitinib therapy with overall survival exceeding 1.5 year. Exploring the toxicity of multi-kinase targeting agents in GISTs may allow better adjusted therapy as well as to define novel pharmacodynamics markers. Primary tumor genotype and sunitinib-induced arterial hypertension (as surrogate of its antiangiogenic activity) are two independent factors influencing the progression-free survival and OS. The mechanism of side effects and its correlation with pharmacogenetic data during sunitinib therapy need further studies.

## Competing interests

P. Rutkowski, C. Osuch, Z. Nowecki and A. Wozniak have received honoraria and travel grants form Novartis, P. Rutkowski served in advisory board for Novartis, J. Kroc is employed by Pfizer; P.Rutkowski and Z. Nowecki have received honoraria and travel grants from Pfizer.

## Authors' contributions

*Study concepts: *PR, EB, AW, JK, ZIN, IL, JL. *Study design: *PR, AW, CO, IL, JL. *Data acquisition*: PR, EB, AK, TŚ, SF, JK, IŁ, WM, CO, AW, UG, ZIN. *Data analysis and interpretation: *PR, EB, IŁ, MB, EM, KW, AW, UG, JS, JL. *Statistical analysis*: PR, IL, EM. *Manuscript preparation*: PR, AW, JL, MB, IŁ. Manuscript review: PR, EB, AK, TŚ, SF, JK, IŁ, MB, WM, CO, EM, KW, AW, UG, ZIN, JS, JL. All authors read and approved the final manuscript.

## Pre-publication history

The pre-publication history for this paper can be accessed here:

http://www.biomedcentral.com/1471-2407/12/107/prepub
